# Culture-Negative Neutrocytic Ascites in a Patient With Cardiac Ascites From End-Stage Heart Failure

**DOI:** 10.7759/cureus.55802

**Published:** 2024-03-08

**Authors:** Kori X Ormachea, Lennon Gregor, Janina Quintero, Bistees George, Sandeep Singh

**Affiliations:** 1 Internal Medicine, Indiana University School of Medicine, Indianapolis, USA

**Keywords:** culture-negative neutrocytic ascities, polymorphonuclear neutrophils, transesophageal echocardiogram (tee), tricuspid valve regurgitation, heart failure with reduced ejection fraction

## Abstract

There are two significant groups of infection regarding ascitic fluid: spontaneous bacterial peritonitis (SBP) and culture-negative neutrocytic ascites (CNNA). SBP and CNNA typically occur in patients with cirrhosis. A 46-year-old male with end-stage biventricular heart failure presented with a heart failure exacerbation. He was treated with intravenous diuretics with the improvement of hypervolemia. He remained hospitalized to undergo an evaluation for tricuspid valve repair, but given the severity of his bi-ventricular heart failure, he underwent a heart transplant evaluation. As part of the work-up, he underwent an abdominal ultrasound that was significant for severe ascites but did not note an abnormal hepatic architecture suggestive of cirrhosis. A liver biopsy was then performed, which confirmed no evidence of cirrhosis. His hospitalization was complicated by refractory cardiac ascites, which required a bi-weekly paracentesis. The serum albumin-ascites gradient (SAAG) from his initial paracentesis was 1.4, indicating the etiology was from portal hypertension. The total protein was greater than 2.5 in multiple studies, so the etiology was less concerning for cirrhosis and secondary to his heart failure. About two weeks into his hospital course, he developed a leukocytosis but remained hemodynamically stable and asymptomatic from an infectious standpoint. Analysis of his ascitic fluid initially was negative for infection, but he later developed an elevated total neutrophil count on a subsequent ascitic fluid analysis study. The body fluid culture remained negative for bacterial growth. Hepatology was consulted, and he met the criteria for CNNA, so treatment with ceftriaxone was initiated. After initiating antibiotics, his leukocytosis and elevated ascitic fluid total neutrophil count resolved. Ascitic infections such as CNNA generally occur in patients with liver cirrhosis but may occur in patients without cirrhosis, as observed in our patient. This case highlights that patients with cardiac ascites can develop ascitic fluid infections that may have an impact on their mortality. The precipitating factor that enabled the patient to develop CNNA is unclear but may be related to the translocation of bacteria during his congestive heart failure exacerbation. Although uncommon in a patient with cardiac ascites, an early diagnosis of CNNA and the initiation of antibiotics can be important in preventing patient mortality.

## Introduction

Liver cirrhosis is the most common cause of ascites in the United States and is responsible for about 80% of cases [1]. Ascites develop from shifts in portal pressure and oncotic pressure. To aid in determining the etiology of ascites, the difference in the serum albumin to the ascitic fluid albumin gradient (SAAG) is often used along with the total protein in the ascitic fluid [2]. A SAAG greater than 1.1 indicates the presence of portal hypertension. Heart failure is a rare cause of ascites and accounts for approximately 3% of cases [1]. The elevated portal pressure from cardiac ascites is due to venous congestion from impaired venous return to the heart.

There are two significant groups of infection regarding ascitic fluid: spontaneous bacterial peritonitis (SBP) and culture-negative neutrocytic ascites (CNNA). Diagnostic criteria for CNNA are an ascitic fluid polymorphonuclear leukocyte count >250/mm, an ascitic fluid culture negative for bacterial growth, no prior antibiotic therapy within 30 days, and no infectious source within the abdomen [3-5]. CNNA generally has a lower risk for mortality, which was demonstrated across multiple studies [5]. However, there is conflicting data between more recent studies reporting mortality comparable to SBP [6,7]. SBP and CNNA generally occur in patients with ascites due to cirrhosis and are rarely observed in patients with ascites due to heart failure. This case highlights that patients with cardiac ascites can develop ascitic fluid infections that may have an impact on their mortality.

Informed consent was obtained from the patient for the publication of this case report and the images included.

## Case presentation

A 46-year-old male, with a history of bi-ventricular heart failure with reduced ejection fraction (EF) due to non-ischemic cardiomyopathy from radiation and chemotherapy for a previous pulmonary sarcoma, obstructive sleep apnea, and ascites, initially presented to an outside hospital with worsening dyspnea and anasarca for the past few days. He was transferred to our care for ongoing management of hypervolemia and evaluation for intervention for severe tricuspid regurgitation. 

**Figure 1:**
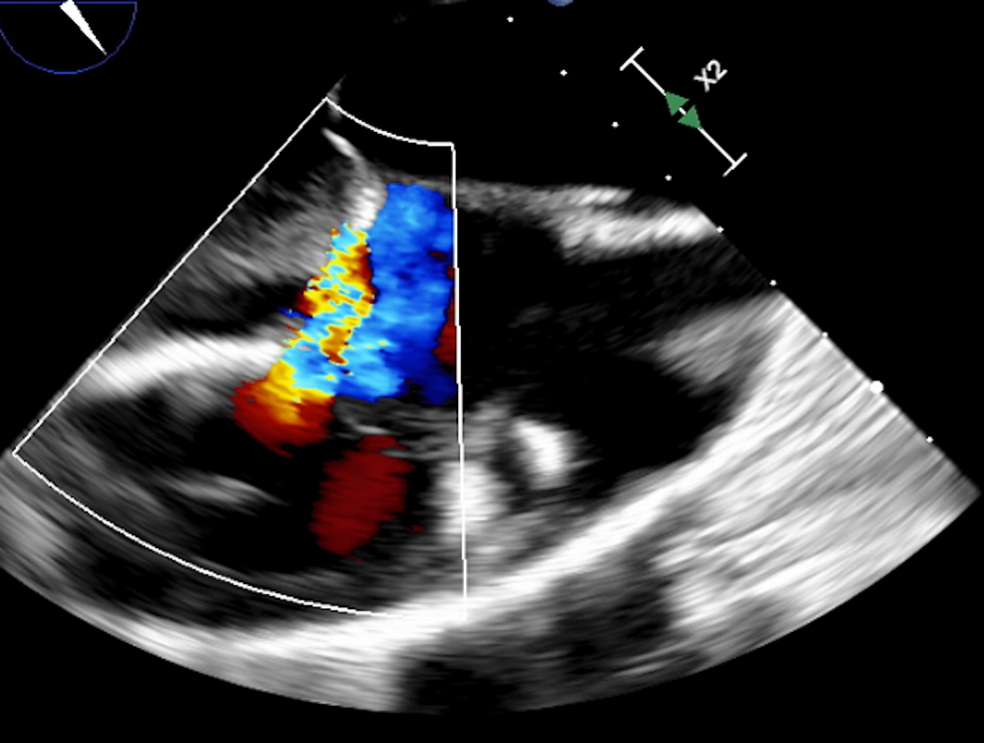
Severe tricuspid regurgitation noted on transesophageal echocardiogram.

Respiratory symptoms and anasarca were present for months but acutely worse over a few days, including new onset orthopnea. The physical exam was notable for jugular vein distention, bilateral crackles, ascites, and 4+ pitting edema bilaterally. Labs were significant for an elevated pro-brain natriuretic peptide at 2,736, a mildly elevated aspartate aminotransferase (AST) at 44, and a low sodium at 130. A transthoracic echocardiogram (TTE) noted an EF of 36%, elevated left ventricular end-diastolic pressure, stage 2-3 diastolic dysfunction, and a normal right ventricle size with mildly reduced systolic function. A transesophageal echocardiogram (TEE) was later performed and noted an EF of 30%, global left ventricular and right ventricular hypokinesis, a patent foramen ovale with bidirectional shunt, and severe tricuspid regurgitation (Figure 1). A chest X-ray was performed and was significant for chronic bilateral lung disease with right lobe fibrosis and concomitant edema bilaterally.

He was initiated on intravenous (IV) diuresis with Lasix 80 mg daily with improvement of his dyspnea and edema. He had persistent hypotension, so he continued on his home midodrine 5 mg three times daily. Regarding his recurrent ascites, he required a paracentesis every five to seven days. He was initially undergoing evaluation for a tricuspid valve repair by the Cardiothoracic Surgical team. Since his bi-ventricular heart failure was advanced, a heart transplant was initiated instead of pursuing a tricuspid valve repair. The Hematology Oncology team was consulted for clearance before the transplant. A computed tomography (CT) of his chest, abdomen, and pelvis was negative for any recurrence of sarcoma, so he was cleared for a heart transplant.

**Figure 2:**
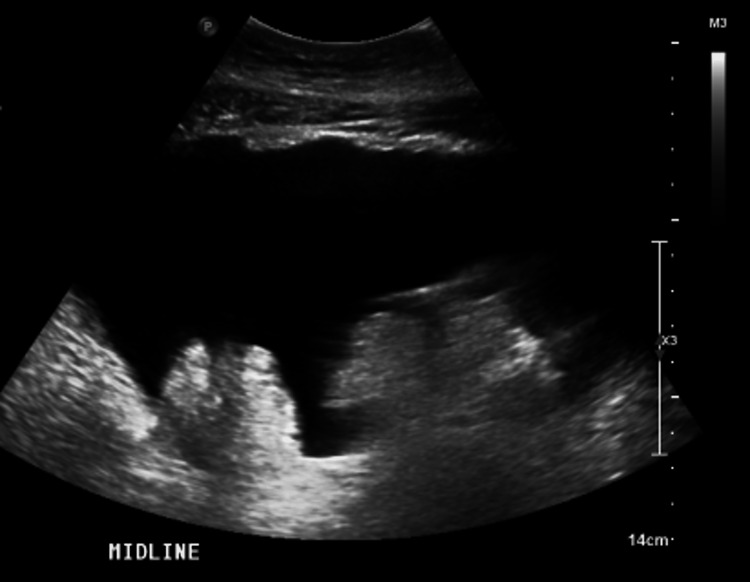
Significant ascites noted on abdominal ultrasound.

Fluid studies from his first paracentesis on admission were negative for infection. The SAAG from his initial paracentesis was 1.4, indicating the etiology was from portal hypertension. The total protein was greater than 2.5 in multiple studies, so the etiology was less concerning for cirrhosis. An abdominal ultrasound with a Doppler was obtained. The findings noted significant ascites with normal hepatic flow but did not demonstrate an abnormal hepatic architecture suggestive of cirrhosis indicating that heart failure was the most likely etiology for his ascites (Figure 2).

On a subsequent paracentesis a week later, his fluid studies noted a total neutrophil count (TNC) of 1,100, but he remained asymptomatic along with negative fluid cultures. The abdominal exam was only significant for ascites without abdominal pain or tenderness. He has hypotension at baseline; otherwise, his vitals remained within normal limits. The only indication that he may have had an underlying infection was a slowly rising white blood cell count (WBC) that peaked at 14.

We consulted hepatology to evaluate for liver cirrhosis and for the elevated ascitic fluid TNC that was culture-negative. The abdominal ultrasound obtained did not have any findings of cirrhosis. A liver biopsy was performed and was significant for stage one and two fibrosis but also negative for cirrhosis. Hepatology recommended empirically treating for culture-negative neurolytic ascitic infection with a seven-day course of ceftriaxone. The TNC was noted to be a downtrend on subsequent fluid studies along with the resolution of his leukocytosis. Additional fluid cultures remained negative after that. He completed the transplant evaluation, including a right heart catheterization (RHC) with a pulmonary artery pressure of 41/23, pulmonary capillary wedge pressure of 27/35, a right ventricular pressure of 38/11, and a right atrial pressure of 29/28. He required an escalation of his diuretic regimen to an IV Lasix infusion at 20 mg an hour, oral metolazone at 5 mg daily, and an IV infusion of milrinone at 0.25 mcg/kg/min. He was listed for heart transplant United Network for Organ Sharing (UNOS) 2 status for worsening heart function based on his RHC.

## Discussion

This case presents a rare complication of cardiac ascites in a patient with advanced, end-stage heart failure. CNNA typically occurs in patients with cirrhosis. This presentation is unique because CNNA appeared in a patient without evidence of cirrhosis observed on abdominal ultrasound and liver biopsy. Cardiac ascites has more protein in the ascitic fluid than ascites that develop from cirrhosis. The lower incidence of SBP in cardiac ascites is thought to be related to the bactericidal effect of protein [8]. Generally, CNNA is less common and less severe than SBP, but data are conflicting regarding the overall risk of mortality [6,7]. It has been postulated that the differences in mortality are due to patients with SBP typically having more advanced cirrhosis. Therefore, those patients can develop life-threatening complications associated with advanced cirrhosis.

In our patient, the ascitic fluid cultures remained negative during his infection. Negative cultures are generally due to organisms that are difficult to culture. Current studies have investigated bactDNA as an alternative to fluid cultures to evaluate for a pathogen to identify patients at risk for ascitic infections [9]. Since the mortality of CNNA is unclear, using an alternative method of diagnosis could allow for earlier intervention with antibiotics in clinical cases such as ours, which was unclear given his lack of symptoms and hemodynamic stability. Other markers that are used for treatment response in CNNA are lymphocyte-to-monocyte ratio (LMR) and C-reactive protein. A study demonstrated the use of LMR and C-reactive protein to monitor treatment response. In the study, after the study group received antibiotic treatment, C-reactive protein levels were significantly lower, and LMR was elevated [3]. We did not use these markers upon initial diagnosis and treatment of our patient’s care of CNNA. The use of these markers could be considered in unclear patient presentations such as our patient’s case.

The approach to treatment involves antibiotic therapy with either an aminoglycoside or a five-day course of ceftriaxone. A study involving 50 patients with CNNA underwent treatment with ceftriaxone for five days. The study outcomes indicated that 78% of patients resolved their infection. Another observation in this study was a low hospital mortality rate of 4% in the group treated with ceftriaxone [10]. Generally, a response to treatment is defined as a 25% reduction in TNC count [11]. Our patient was treated with ceftriaxone with improved TNC count on subsequent paracenteses, and his WBC returned to a normal range.

Refractory ascites occurred in our patient due to poor cardiac output from biventricular heart failure. This resulted in a weekly to bi-weekly paracentesis. Ascites is a complication of congestive heart failure, especially when involving elevated right heart pressures. However, an ascitic fluid infection is not a recognized complication of heart failure, but chronic end-stage heart failure may have contributed. A case report involving a patient with cardiac ascites who presented with a congestive heart failure exacerbation and concurrent viral colitis developed SBP. Investigators hypothesized that viral colitis promoted the translocation of bacteria, which may have contributed to the development of SBP [12]. Another contributing factor to our patient was that he presented with an acute heart failure exacerbation. During a congestive heart failure exacerbation, bacterial and fungal translocation can be precipitated by disruption of the intestinal barrier from intestinal hypoperfusion and congestion from excess volume [13]. The precipitating factor in our patient is unclear, but this case highlights that ascitic infections such as CNNA can occur in patients with ascites from advanced heart failure.

## Conclusions

Ascites from a cardiogenic cause such as heart failure is uncommon and account for approximately 3% of cases. Ascitic infections such as CNNA generally occur in patients with liver cirrhosis but may occur in patients without cirrhosis, as observed in our patient. This case highlights that patients with cardiac ascites can develop ascitic fluid infections that may have an impact on their mortality. Although uncommon, an early diagnosis of CNNA and the initiation of antibiotics can lead to more favorable outcomes.
